# Role of Staging Laparoscopy in Patients Undergoing Pancreaticoduodenectomy

**DOI:** 10.7759/cureus.5906

**Published:** 2019-10-14

**Authors:** Mohammad I Ashraf

**Affiliations:** 1 Surgical Oncology, Shaukat Khanum Memorial Cancer Hospital and Research Center, Lahore, PAK

**Keywords:** pancreaticoduodenectomy, pancreatic carcinoma, periampullary tumor, staging laparoscopy

## Abstract

Background

Pancreatic cancer patients undergoing curative resection need staging laparoscopy for detecting metastatic disease not picked up on standard radiological scans. Identifying these patients can help to prevent unnecessary laparotomies and early induction of palliative therapies.

The aim of our study was to determine the effect of staging laparoscopy on resectable pancreatic or periampullary tumors at our hospital.

Methods

Patients recommended pancreaticoduodenectomy between September 2014 and June 2018 were included in this study. Any significant finding and its impact on management was recorded.

Results

A total of 120 patients underwent staging laparoscopy. Fifteen patients had suspicious lesions and one patient had cirrhotic liver on staging laparoscopy. Out of these 15 patients nine patients had liver lesions, three patients with peritoneal nodules and three patients having both liver and peritoneal lesions. Among patients with liver lesions, four patients were found to have metastatic deposits. All of the remaining lesions were benign. Plan of curative resection was changed in five patients. Three patients who were planned for pancreaticoduodenectomy cancelled because of hepatic metastasis. One patient with hepatic metastasis showing neuroendocrine tumor considered for resection. One patient with resectable disease had cirrhotic liver on laparoscopy, so not medically fit for this major surgery.

Conclusion

In patients planned for pancreaticoduodenectomy, staging laparoscopy is an important step to detect metastatic disease involving peritoneum or viscera. It is very helpful in determining the stage of disease and further management plan.

## Introduction

Seventh leading cause of cancer-related deaths worldwide is pancreatic cancer [1]. It is the third most common cause of cancer-related mortality in the USA. Only 15-20% patients have resectable disease at presentation while remaining patients have either locally advanced or metastatic disease making them unsuitable for surgical resection [2]. Nonspecific symptoms on early stages, limited diagnostic tests available for detection and limited response of pancreatic cancer to chemotherapy and radiotherapy are responsible for overall grave outcome of pancreatic cancer [3-5]. For patients with locally advanced disease, the median survival is approximately 12 to 15 months [6]. Overall five-year survival is about 8.3% [7].

The poor outcome of pancreatic cancer is due to late detection and inadequate staging of the disease [8]. So exact staging of pancreatic cancer is necessary for proper management. Different imaging modalities used for staging include ultrasound, CT scan, magnetic resonance imaging, endoscopic ultrasonography, endoscopic retrograde cholangiopancreaticography and positron emission tomography scan [9]. CT scan is best initial imaging modality but still 41% patients with resectable disease on CT scan were found to have irresectable disease on surgical exploration leading to unnecessary laparotomies [10].

Pancreaticoduodenectomy with adjuvant chemotherapy at present is only curative treatment present for pancreatic and periampullary tumors [11]. Unfortunately only 15-20% patients have resectable disease on presentation [2]. This is a cause of grave concern for the patients and the surgeons. There is undue burden on the hospital if a planned resection becomes unresectable. This is where the role of staging laparoscopy before proceeding with formal surgical resection has come in. A diagnostic laparoscopy before the procedure avoids an unnecessary laparotomy and prevents the patient from perioperative morbidity and mortality. Staging laparoscopy is now being preferred by most surgeons as a part of initial staging protocol [12]. Now it has become a gold standard to perform staging laparoscopy before formal exploration despite the advent of newer imaging modalities.

The aim of our study was to determine the effect of staging laparoscopy on resectable pancreatic or periampullary tumors at our hospital. It will reduce unnecessary laparotomies in patients having peritoneal and liver metastasis.

## Materials and methods

The study was conducted at Shaukat Khanum Memorial Cancer Hospital and Research Center (SKMCH & RC) in which patients according to sample size were reviewed from September 2014 to June 2018 after taking approval of IRB. Endoscopic ultrasonography (EUS) and CT scan were main imaging modalities for detection and staging of these lesions.

The exclusion criteria were venous occlusion or encasement of portal or superior mesenteric artery, vein or celiac trunk, duodenal obstruction, considerable impairment in normal functioning and serious comorbidity. Patients who had radiological-based irresectable disease either having locally advanced disease involving vitals structures or metastasis were excluded from this study. All other patients with resectable disease were included in our study.

A standardized technique of staging laparoscopy was devised to be performed in all patients. Abdominal cavity was approached via open technique through infraumbilical incision. Working port was placed under direct vision to help in visualization of abdominal organs. No disruption of lesser sac was done. Biopsies were performed using laparoscopic L-shaped hook and sent for histopathological assessment.

The data of qualitative variables like gender, site of tumor, T stage, N stage of tumor, suspicious lesions, histopathology of suspicious lesions, management and change in management plan were collected. The quantitative variables like age, CA19-9 levels were also collected. The data organised and entered in IBM SPSS 21 (IBM Corp., Armonk, NY) and analysed by using the statistical tools.

## Results

A total of 120 patients who had radiologically resectable disease underwent staging laparoscopy. Out of these 72 were males and 48 were females. Mean age of the patients was 58 years as depicted in Table 1.

**Table 1:** Demographics and characteristics

Variables		Values	Percentage
Gender	Male	72	60
	Female	48	40
Age (Median)		58	
CA 19-9 (Median)		98.5	
Site of Tumor	Pancreatic	79	65.8
	Periampullary	41	34.2
T Stage	T1	11	9.1
	T2	63	52.5
	T3	39	32.5
	T4	07	5.83
N Stage	N1	45	37.5
	N2	61	50.8
	N3	14	11.7

Fifteen patients had suspicious lesions and one patient had cirrhotic liver on staging laparoscopy. Out of these 15 patients nine patients had liver lesions, three patients had peritoneal nodules and three patients had both liver and peritoneal lesions. Among patients with hepatic lesions, three patients were found to have metastatic deposits of pancreatic cancer while one had metastasis of periampullary neuroendocrine tumor. All of remaining lesions were benign. Plan of curative resection was changed in five patients as the result of staging laparoscopy. Three patients who were planned for pancreaticoduodenectomy cancelled because of hepatic metastasis. One patient with hepatic metastasis showing neuroendocrine tumor considered for resection. One patient with resectable disease had cirrhotic liver on laparoscopy and hence curative resection was not undertaken in him as depicted in Table 2.

**Table 2:** Laparoscopic findings and management

Variables		Values	Percentage
Suspicious Lesions	No	105	87.5
	Yes	15	12.5
Histopathology of suspicious lesions	Benign	11	73.3
	Malignant	4	16.7
Management	Whipple’s procedure	89	74.1
	Bypass	21	17.5
	No surgical intervention	10	8.4
Change in Management	No	115	95.8
	Yes	5	4.2

## Discussion

Use of staging laparoscopy for pancreatic cancer is a major step in management of pancreatic and periampullary tumors. It can help to visualize the occult disease not detected by radiology. Thus, staging laparoscopy has the potential to prevent unnecessary laparotomies and associated morbidity and mortality. It is also cost effective if performed before definite surgery [13]. About 41% patients who were deemed resectable on imaging found to have local spread, peritoneal disease or metastatic visceral disease making them irresectable [10]. Simple laparoscopy with peritoneal and visceral biopsy can help us to detect otherwise undetectable disease. Nonoperative palliative modalities such as stenting can be used in these patients who have poor functional status and poor prognosis [14]. Frozen section for evaluation of biopsy taken by staging laparoscopy can be followed by resection under same anesthesia if biopsy is negative. This method has reduced the unnecessary laparotomies from 24 to 44% [15]. Staging laparoscopy not only prevents unnecessary exploration but also leads to early induction of chemotherapy and better overall survival as compared to those when underwent exploration [16].

**Figure 1:**
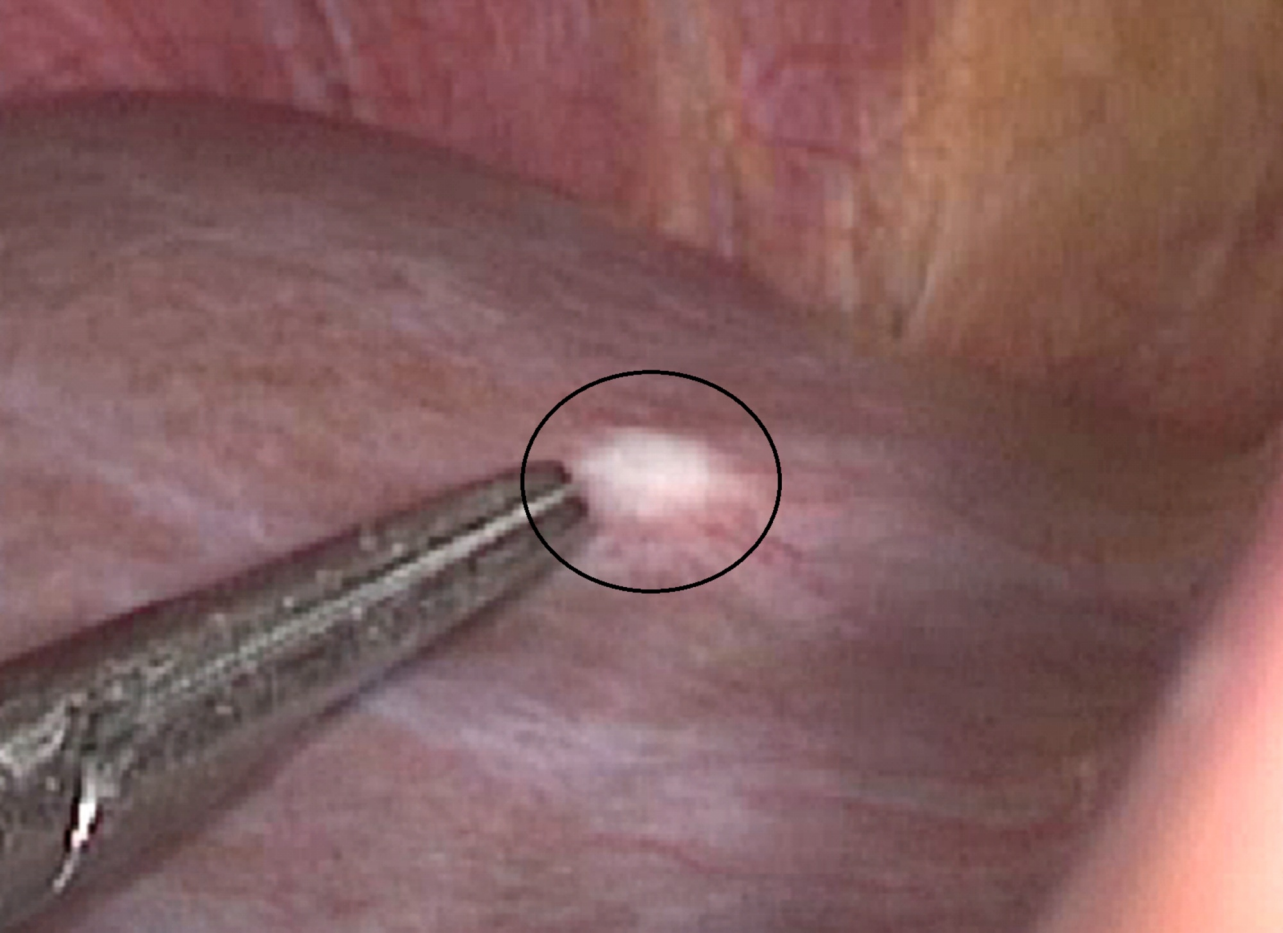
Hepatic metastasis from pancreatic adenocarcinoma

In our study, 12.5% (15/120) patients who were resectable on radiological imaging were found to have suspicious lesions on staging laparoscopy and further evaluated by taking biopsy. Out of 15 patients, four patients' (26.7%) suspicious lesions were metastatic. Out of these four patients, three patients had metastatic pancreatic adenocarcinoma, so plan of curative resection was deferred and these patients were referred for palliative chemotherapy (Figure 1).

**Figure 2:**
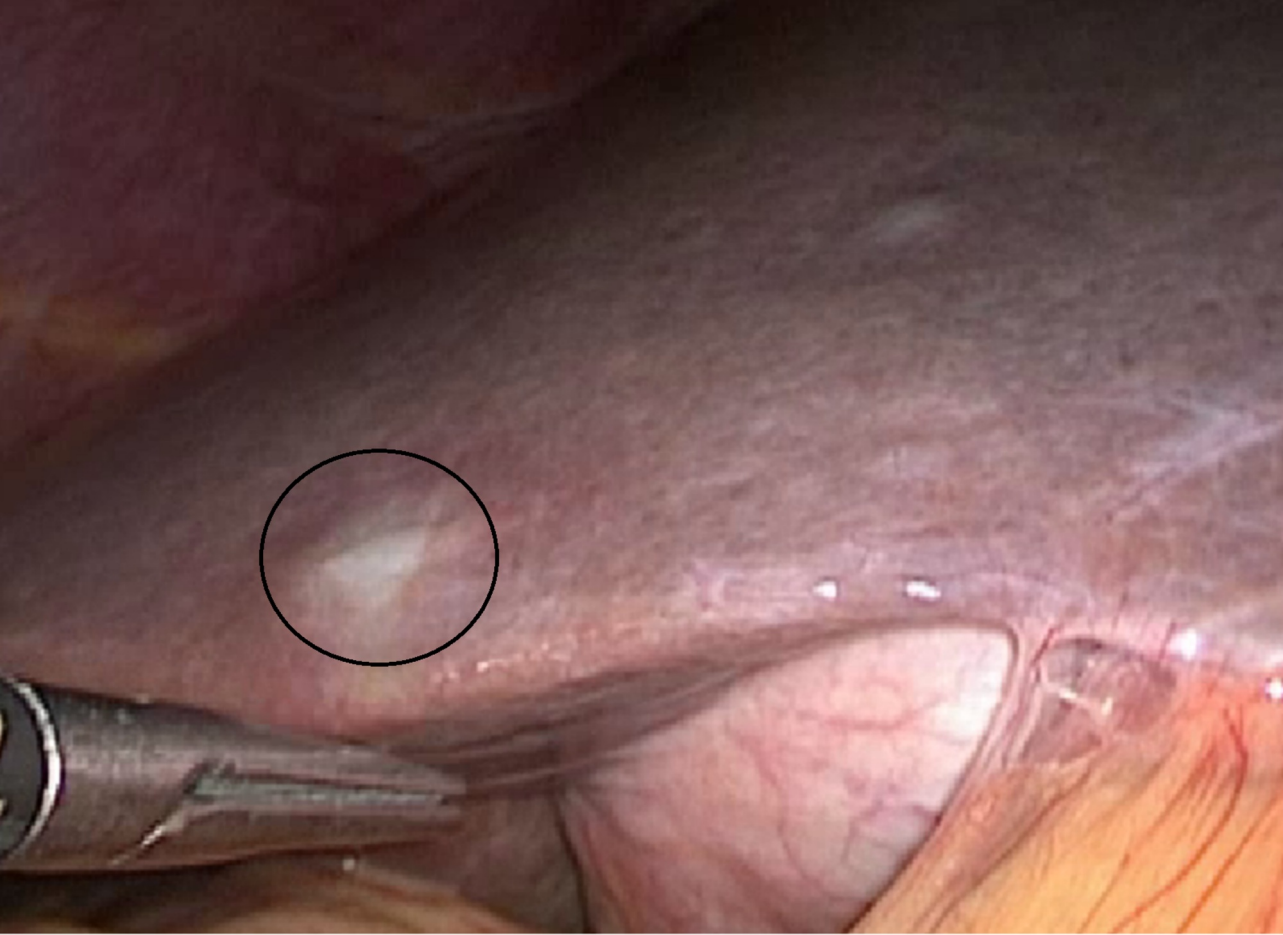
Hepatic metastasis from periampullary neuroendocrine tumor

While one patient had metastatic neuroendocrine tumor with primary lesion of periampullary region. In this patient, curative resection, i.e., Whipple’s procedure with resection of metastatic lesion was done in single setting (Figure 2).

Moreover, one patient who was fit clinically and resectable from radiological point of view was found to have cirrhotic liver on laparoscopy and curative resection was deferred due to high chance for post-operative hepatic failure in this patient.

This is concurrent with the study performed by Allen et al. [10]. He concluded that staging laparoscopy reduced the rate of non-resectable laparotomies in patients who were found to have resectable pancreatic or periampullary carcinoma on CT scan. On average, 21 unnecessary laparotomies in 100 people in whom resection with curative intend was planned could be avoided by using laparoscopic staging before the procedure.

In our study curative resection, i.e., Whipple’s procedure was performed in 89 patients only. While remaining patients were found to have locally advanced disease which was not suitable for resection on exploration. Out of these 31 patients, bypass either double or triple was done in 21 patients. No surgical intervention was done in 10 patients besides exploratory laparotomy.

Management plan was changed in 4.2% (5/120) patients due to staging laparoscopy. Although laparoscopy is useful in preoperative staging and management decision in patients with pancreatic or periampullary tumors but still there were 31 patients (25.8%) who were resectable on initial staging but were found to be irresectable when operated from curative intent.

Meta-analysis conducted by Ta et al. included 15 studies [12]. In 12 studies staging laparoscopy showed irresectable disease in 350 (20% ) out of 1750 patients who had resectable disease radiologically. In remaining three studies on 242 patients, 86 (36%) patients found to have metastasis on staging laparoscopy not detected by radiological scans. This meta-analysis showed that staging laparoscopy had a definite role in detecting radiologically occult disease which influenced the final management plan.

So staging laparoscopy is useful in detecting and evaluating the suspicious visceral or peritoneal lesion but it is not very helpful in accessing the localized advancement of disease. But its use in combination with CA 19-9 levels can be very useful in detecting true stage of the disease and advising proper management plan.

The limitation of our study was that it was a single centre study (SKMCH & RC) which is one of the two JCI-accredited institutions in Pakistan. Hence to make the findings of the research more generalizable and reproducible this study should be conducted in multiple centres. It would bring validity to the results of this very important problem.

## Conclusions

In patients planned for pancreaticoduodenectomy, staging laparoscopy is an important step to find hepatic or peritoneal metastasis. Biopsy of any suspicious lesion if found during staging laparoscopy is very helpful in determining the nature of disease and further management plan. Staging laparoscopy avoids unnecessary exploration and hence post-operative morbidity can be avoided in patients who were found to have metastatic disease. Also these patients can be referred early for chemotherapy.
